# Making pragmatic choices: women’s experiences of delivery care in Northern Ethiopia

**DOI:** 10.1186/1471-2393-12-113

**Published:** 2012-10-19

**Authors:** Tesfay Gebrehiwot, Isabel Goicolea, Kerstin Edin, Miguel San Sebastian

**Affiliations:** 1 Department of Public Health, College of Health Sciences, Mekelle University, Mekelle, Ethiopia; 2 Department of Public Health and Clinical Medicine, Epidemiology and Global Health, Umeå University, Umea, Sweden

## Abstract

**Background:**

In 2003, the Ethiopian Ministry of Health launched the Health Extension Programme (HEP), which was intended to increase access to reproductive health care. Despite enormous effort, utilization of maternal health services remains limited, and the reasons for the low utilization of the services offered through the HEP previously have not been explored in depth.

This study explores women’s experiences and perceptions regarding delivery care in Tigray, a northern region of Ethiopia, and enables us to make suggestions for better implementation of maternal health care services in this setting.

**Methods:**

We used six focus group discussions with 51 women to explore perceptions and experiences regarding delivery care. The data were analysed by means of grounded theory.

**Results:**

One core category emerged, ‘making pragmatic choices’, which connected the categories ‘aiming for safer deliveries’, ‘embedded in tradition’, and ‘medical knowledge under constrained circumstances’. In this setting, women – aiming for safer deliveries – made choices pragmatically between the two available models of childbirth. On the one hand, choice of home delivery, represented by the category ‘embedded in tradition’, was related to their faith, the ascendancy of elderly women, the advantages of staying at home and the custom of traditional birth attendants (TBAs). On the other, institutional delivery, represented by the category ‘medical knowledge under constrained circumstances’, and linked to how women appreciated medical resources and the support of health extension workers (HEWs) but were uncertain about the quality of care, emphasized the barriers to transportation.

In Tigray women made choices pragmatically and seemed to not feel any conflict between the two available models, being supported by traditional birth attendants, HEWs and husbands in their decision-making. Representatives of the two models were not as open to collaboration as the women themselves, however.

**Conclusions:**

Although women did not see any conflict between traditional and institutional maternal care, the gap between the models remained and revealed a need to reconcile differing views among the caregivers. The HEP would benefit from an approach that incorporates all the actors involved in maternal care, at institutional, community and family levels alike. Reconsideration is required of the role of TBAs, and a well-designed, community-inclusive, coordinated and feasible referral system should be maintained.

## Background

Despite the international emphasis since the 1990s on the need to address the unmet health needs of pregnant women and children, progress in reducing maternal mortality has been slow. This is particularly worrying in sub-Saharan Africa where more than 300,000 women still die each year during pregnancy and childbirth; most of them die because they lack access to skilled delivery attendance and emergency care [1-5].

In the international arena, maternal mortality reduction policies and programmes existed well before 1987, when the Safe Motherhood Initiative was launched, but their focus has been shifting over the years, influenced not only by new emerging evidence but also by competing interests [4,6]. During the 1970s and 1980s the focus was on training traditional birth attendants (TBAs) and community health workers. After many studies confirmed that training TBAs was not cost-effective when compared with the provision of professional skilled care [7,8], training programs for TBAs were discouraged, as well as other community-based actions. Instead, programmes modified their focus to skilled delivery care and the implementation of emergency obstetric care services within health facilities [4,6,9,10]. Despite noteworthy achievements, focusing on the implementation of emergency obstetric care did not automatically result in women’s better access to services [6,9,11]. Currently, approaches such as the ‘Partnership for maternal, newborn and child health initiative’ from the World Health Organization (WHO) advocate for an integrative approach to maternal health care considering two key aspects of the continuum of care: (1) ensuring maternal health needs with a life cycle approach, and (2) providing maternal health care at the household, community and institutional level. In order to reduce maternal mortality all these actors should be mobilized to work together [12,13].

Although no universal recipe for scaling-up maternal and child health interventions exists, the architecture of the health system and the local social context must be understood [6,9,14]. Accordingly, to decrease maternal mortality and morbidity, countries need strong health systems that match care provided at the household and community levels. The health service should provide accessible, available, acceptable and good-quality family planning, abortion and antenatal care together with skilled delivery and postpartum care. All the reproductive health services should be connected to responsive and accountable emergency obstetric services for consultation, transportation and referral [9,12]. After more than thirty years of academic debates about the most effective interventions for maternal mortality reduction, the research community is currently urging a stronger focus on research that explores how to implement these interventions in specific contexts [9,11]. To this end, the perspective of the potential users of these interventions becomes especially relevant.

### Maternal health care in Ethiopia

With a maternal mortality ratio of 673 per 100,000 live births and 19,000 maternal deaths annually, Ethiopia is a major contributor to the worldwide death toll of mothers. Although better achievement was reported with regard to reducing infant and child mortality in the country, slow progress has been achieved in terms of the Millennium Development Goal (MDG) 5, the cornerstone of maternal health [15].

One of the key targets of the MDG5 is to achieve universal access to reproductive health by 2015. In order to fulfil this objective, the Ethiopian Ministry of Health launched a community-based healthcare system in 2003, the Health Extension Programme (HEP), rooted in a primary health care approach.

The HEP is designed to improve equitable access to preventive essential health interventions through community-based health services and to achieve significant basic health care coverage in the country through the provision of a staffed health post to serve an area of approximately 5,000 to 7,000 people (a *kebele*), the lowest administrative unit. Each *kebele* has one health post where two HEWs, after completion of one year’s training, are employed to provide preventive, promotive and basic curative health services to the community. The HEWs deliver healthcare services both at the health post and in the community, with strong focus on sustained preventive health actions and increased health awareness; they provide antenatal care, and may attend deliveries, although whenever a complication emerges, they have to refer to the health centre which is a walk of two to three hours. They are also in charge of supervising TBAs and other voluntary community health workers, who are expected to support health education activities in the communities [8,16,17]. The HEP has been implemented throughout Ethiopia and by May 2008 there were 24,500 trained and deployed HEWs, some 82% of the 30,000 target of the Ministry of Health for 2010/11 [18]. Regarding maternal health, HEWs are expected to provide post-abortion care, family planning, antenatal care, delivery attendance (including referral of obstetric complications) and postnatal care. Basic and comprehensive emergency obstetric care should be available at health centres and hospitals, and a strong referral system that links health posts is expected to mainstream communities’ networks through higher resolution levels [16].

The HEP has showed some achievements in terms of vaccination coverage, family planning, latrine coverage, personal hygiene, environmental sanitation and increased awareness of health benefits at community level [18]. However, in terms of maternal health, universal access to services remains limited, particularly skilled delivery attendance. In 2009, a survey conducted in four regions of Ethiopia reported 32% of women using modern contraceptives, 54% attending antenatal care, but only 9% delivering institutionally [19]. Women with certain characteristics – such as being married, having secondary education and having a history of obstructed labour – have been identified as the most important predictors of preference of skilled birth attendants; however, the reasons for the low utilization of the maternal health care services offered through the HEP previously have not been explored in depth [20].

This study aims to explore women’s experiences and perceptions regarding delivery care in the northern region of Tigray, Ethiopia. By presenting the women’s point of view, our goal is to point out suggestions for better implementation of maternal healthcare services in this setting.

## Methods

### Study area

The study was conducted from September 2010 to January 2011 in two rural districts of Tigray province, Ganta-afeshum and Kilte-awlaelo. These districts are located in the northern region of Ethiopia, more than 800 km away from the capital Addis Ababa. The total population of the two districts in 2007 was estimated to be 188,384 [21].

The two districts included in this study encompass 29 health posts, 10 health centres and two hospitals; around 58 HEWs work in the area. Data from the Tigray Health Bureau have estimated the antenatal care coverage in these two districts to be 52.8% and 80% respectively, whereas skilled delivery attendance drops to 21 and 20% [22].

### Participants

For this study, women who had given birth in the last three years (regardless of their current pregnancy status) were invited to take part in focus group discussions (FGDs). The three year period was chosen in order to have enough eligible participants without too long of a recall period. We included both women who had delivered at home and women who had delivered at health facilities, since we expected their experiences and attitudes to be different. Fifty-one women participated in the FGDs; 27 had delivered at home and 24 at a health institution and their age ranged from 15 to 40 years. The participants differed in terms of parity and educational level, all of them were married and the majority were engaged in farming activities.

Women who had been working as community health volunteers were not included in the FGDs, since they were expected to be more aware of the subject in focus (from training and workshops about maternal health). In order to gather different experiences, FGDs were held both with women who lived in *kebeles* that were close to town and with those located in remote areas.

### Data collection

The HEWs identified potential participants and invited them to come to the health post for the FGD. Once the women arrived, they were requested to choose the place for discussion. The majority of FGDs were conducted outside the health posts. In order to ensure that the women discussed topics more openly, the HEWs and the district health office workers were not allowed to take part in the FGDs.

The first author (TG) moderated all the FGD, and a note-taker was always present as well. Six FGDs were conducted and each lasted between 90 and 120 minutes. Oral informed consent to participation in the recorded FGDs was obtained from every woman. Confidentiality and privacy were guaranteed, names and other information that would enable participants’ identification being removed.

At the beginning, the moderator explained the general topic of the FGD and encouraged the participants to express their ideas freely. The FGD guide included semi-structured open-ended questions with certain key topics to be covered: reasons for women seeking and not seeking antenatal care (ANC) and delivery care (DC), the role of men and relatives in decision-making processes, and encouraging and discouraging reasons to give birth at home and at a health facility (HF). Relevant issues that emerged were followed up in subsequent discussions.

All the FGDs were conducted in Tigrigna, which was the mother tongue of the moderator, the note-taker and the participants. The FGDs were recorded and transcribed verbatim. Handwritten notes were reviewed to add information while we listened to the recordings. The transcriptions were translated into English and thoroughly double-checked against the original by the first author.

### Data analysis

During the whole process of data collection and analysis, memos were recorded to capture ideas and reflections. The translated transcriptions were imported to software for managing qualitative data (Open Code). The data was analyzed informed by a grounded theory approach with the constant comparison method [23]. First, open coding was conducted and codes were negotiated between the authors. Through selective coding the categories and subcategories were refined and the core category was identified.

### Ethical considerations

The study received ethical approval from the University of Mekelle, Ethiopia. Permission was obtained from the district health authorities, besides oral informed consent from participants.

## Results

### Making pragmatic choices

During the data analysis, one core category emerged: ‘making pragmatic choices’. The core category represented how women in this setting were ‘aiming for safer deliveries’, making choices between the two available models of childbirth: home delivery, represented by the category ‘embedded in tradition’, and institutional delivery, represented by the category ‘medical knowledge under constrained circumstances’. Women recognized the risks associated with delivery, acknowledged the positive and negative aspects of both available models, and were pragmatic in their decisions, to the limited extent that the models allowed the mixing of components. Husbands were perceived as supporting women in their aim for a safe delivery.

**Figure 1:**
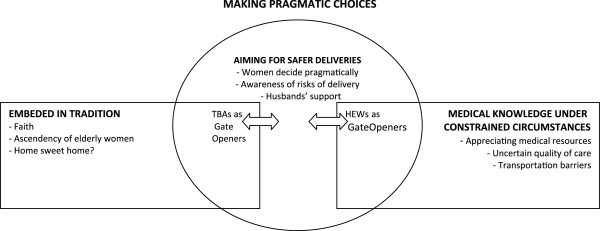
A model showing the relationship between core category “making pragmatic choices” and the categories.

In Figure 1 the rigidity of both models is symbolized by rectangular forms, whereas the pragmatism of women, who did not see a conflict in mixing components of both models, is symbolized by the circular form representing the category ‘aiming for safer deliveries’. Both models included gate-openers who gave support, were close to the women and also appreciated by them, TBAs for the first model and HEWs for the second one (see Figure 1).

### Aiming for safer deliveries

The category ‘aiming for safer deliveries’ represents how women in Tigray acknowledged the risks associated with pregnancy and delivery, and made attempts – supported by their husbands – to enhance their safety during these periods. Within this category, three subcategories emerged: ‘awareness of risks of delivery’, ‘women decide pragmatically’, and ‘husbands’ support’.

### Awareness of risks of delivery

Participants clearly pointed out that they did not consider pregnancy and delivery as illnesses. They perceived delivery as a natural process, an ordinary event that has been managed at home for generations. Previous non-complicated home deliveries further helped to reinforce this perception of delivery as a non-illness process. Women also recognized clearly, however, the risks associated with delivery. They recognized a number of complications that can occur during delivery, namely excessive bleeding, prolonged labour or obstructed labour. Excessive bleeding was recognized as a particularly severe threat.

"‘If it is at home, it is a problem. Nobody will notice if I have a bleed or other complications. They just tell you to keep quiet; I am afraid of losing blood and of a narrow pelvis when giving birth at home’. (Woman who delivered at home - FGD 5) "

When complications happened, women thought that there was little that could be done at home or by TBAs; they considered health facilities to be better prepared to deal with them. Women also believed that prenatal check-ups conducted in health facilities could serve to prevent complications during delivery, thus reducing the risks of home delivery.

"‘Delivering at the hospital has many advantages. They treat complications like bleeding, they give fluid if the mother is exhausted and they also give medication that assists the mother to push down if it is necessary’. (Woman who delivered at health facility - FGD 6)"

### Women decide pragmatically

Women made decisions in a pragmatic way, trying to make the best of the situation and the resources that were accessible to them. They recognized that both home and institutional deliveries had beneficial aspects and limitations. In regards to each of the models, participant women did not feel any contradiction about embracing what they perceived to be good, i.e. they believed that prenatal check-ups were beneficial, but might deliver at home owing to transportation difficulties, and might resort to a health facility if complications such as bleeding, retained placenta or prolonged labor were encountered. In the same way, God’s support during delivery was perceived as available anywhere, independently of the place of delivery; as one participant pointed out, ‘*there is no conflict between God and hikimina [health facility]*’ (Woman who delivered at home - FGD 4).

"‘God is everywhere. Some mothers deliver at home without any help with the help of Saint Mary, that is why I choose to stay at home to give birth whenever conditions seem to be safe, and I also visit hikiminaa for pregnancy check-ups because the health post is close and the HEWs have a good reputation, but for giving birth we decide to go to hikiminaa, when there seems to be any danger because I believe there is a remedy, so, both hikiminaa and God are the same, hikiminaa can also be considered as Saint Mary’. (Woman who delivered at health facility - FGD 3) "

Women were ambivalent, perceiving benefits and drawbacks, both on home delivery and institutional delivery, making it difficult to specify which women or in which cases institutional delivery would be preferred. Women were also realistic, in the sense that even if they considered institutional delivery to be better when complications emerged, they recognized that existing barriers may discourage its use.

### Husbands' support

Women described how their husbands encouraged them during pregnancy check-ups and delivery. Women perceived their husbands as supporters and facilitators of institutional delivery.

"‘If I get sick, my husband intends to take me to the HF. I told him that I wanted to give birth at home, because I was afraid of HFs because of the instruments. Then he encouraged and persuaded me, then they took me to the HF. I gave birth in a nice situation, now I understand HF is good’. (Woman who delivered at health facility - FGD 3) "

Though the decision-making power of husbands ranked below elderly women in the family, participants remarked that husbands played a major role, advocating for institutional delivery, and consequently arranging for transportation.

### Embedded in tradition

The category ‘embedded in tradition’ referred to how women in Tigray were strongly influenced by cultural factors that encouraged them to continue with the traditional model of home delivery. Within this category four subcategories were found: ‘faith’, ‘ascendancy of elderly women’, ‘home sweet home’, and ‘TBAs as gate-openers’.

#### Faith

Faith played a strong role during delivery. The participants described how they trusted God, and especially Saint Mary, for a safe delivery. Furthermore, women described that God was above all and responsible for their destiny. A sense of fatalism was perceived in the way they acknowledged that anything, including complications and death during delivery, could happen at any time, following God’s will. In that sense, what was left to the women and their relatives was to attempt to influence God’s will in a favourable way. Participants referred to the use of prayers during delivery, especially by the elderly people in the household.

Participants also described how relying on God and Saint Mary could give a false sense of safety, preventing women and relatives from taking other actions in order to manage complications in time. Some women said that by relying on prayers for a safe delivery, expectant mothers might have to wait too long before other actions were taken when complications such as bleeding and delayed labour occurred at home:

"‘Well, it would be good if I visit the health facility, but owing to the backward tradition, I do not even call on HEWs during labour, there are few women who argue for calling in HEWs, as they say “St. Mary will support me, no need for HEWs”; this is the old tradition’. (Woman who delivered at home - FGD 3)"

#### Ascendancy of elderly women

Participants described how decisions regarding where to deliver, whom to call and when and where to seek help when complications emerged were strongly influenced by elderly women in the household, namely the mothers or mothers-in-law of the pregnant women.

During the FGDs, women observed that expectant mothers were pressured by their old parents and relatives to give birth in the same way as previous generations did, at home. Obedience to elderly relatives and parents was expressed as a powerful force for women, even if they did sometimes defy them.

"‘My grandparents wanted me to wait a little longer at home. They told me to wait and I gave birth incidentally. Generally elderly women influence you to stay at home; however, some women obey the order and give birth at home and others prefer to go the health facility and give birth there’. (Woman who delivered at home - FGD 5) "

Participants stated that whenever the issue of visiting a HF was raised, elderly women became worried, because they believed that attendants and relatives were not allowed in the delivery room.

Participants also revealed how elderly women distrusted health facilities and portrayed them as risky for mothers and newborns. In health facilities there are frequent check-ups which uncover their bodies, and expose them undressed: unacceptable according to cultural norms. Moreover, elderly women may also argue that at health facilities mothers-to-be may be dangerously exposed to cold (e.g. the windows are open and women are told to remove their clothes and wear light gowns), which they believe delays uterine contraction and extends labor.

However influential these elderly women might be, participants also expressed their disagreement with some of the harmful traditions that have been practised by elderly women during home delivery. For example, they described how elderly women have been facilitating breast-feeding of newborn babies by women other than their mothers, which they thought could expose them to HIV infection. They recalled how newborn babies were offered butter, water and sugar soon after delivery by elderly women, which they thought might produce severe abdominal cramps. Participants also expressed their dislike of the tradition of putting dung on the umbilicus of newborn babies.

Women expressed that accessing ANC was woman’s decision; however, regarding delivery care the decision making was much more dependent on others. Women reported that husbands were in favor of institutional delivery, whereas, the elderly favored home delivery. Women also revealed that when emergency care was sought, community members called HEWs. When HEWs were called, they became in charge, since they are responsible and accountable for any health event encountered at the community level.

#### Home Sweet Home?

This subcategory referred to how women perceived home as a convenient and familiar place for delivery. At home women felt warm, and received the love and support of all family members. When they delivered at home, they did not have to worry about transportation, costs, opening times or availability of staff.

Another argument in favour of home delivery referred to the domestic tasks that were considered as women`s responsibility, such as caring for the rest of the children. Participants said that they preferred to stay at home where they were able to carry out domestic activities, better than travelling to the health facility and staying there for a long time. Women considered themselves the only responsible family members to engage in domestic activities, and worried that if they left the home for a long period, such as for delivery, these tasks would be neglected.

"‘I stayed at home to look after my children. I should manage my life at home, I have kids to take care of. I am the one who gives them whatever they need, food, drink and the like’. (Woman who delivered at home - FGD 2)"

Women considered home as a familiar environment, and they perceived delivering at home as surrounded by known people, love and good hospitality. However, women also acknowledged the disadvantages of home delivery; e.g. home delivery may not always be safe, while at the health facility health workers could keep an eye on expectant mothers and intervene if complications emerged.

"“I am not sure, it is not good to stay at home. I don’t think home delivery is safe. Rather when you deliver at health institution the HWs keep an eye on you and take good care of you. They are responsible.” (Woman who delivered at health facility - FGD 5) "

Challenging opinions also emerged, in that expectant mothers would not be able to engage in any activity during labour or postpartum. Thus, the argument for encouraging home delivery as a way of enabling women to continue fulfilling domestic tasks was considered unreasonable, as one participant from Beati expressed it:

"‘How I would be able to look after my family during labour? It is difficult to look after your family at the time of labour. And even when someone delivers at hospital they come back home on the second day. Therefore it is only because of backward tradition. I give birth at home only because I have never felt pain or got sick’. (Woman who delivered at home - FGD 4)"

#### TBAs as gate-openers

Women expressed their closeness to TBAs, who were their grandmothers or other relatives, such as aunts or mothers-in-law. TBAs were perceived as strongly attached to the participant women and their communities, and highly influential on the decision regarding place of delivery.

Women said that TBAs advocated home delivery, but they were not completely antagonistic to medical knowledge; they integrated some medical supplies in their practice, and they were perceived as willing to refer complicated cases to the health facilities. TBAs did not oppose referral to health facilities when complications emerged. When they believed the delivery was unmanageable or complicated, TBAs were perceived as willing to refer to health facilities. Women viewed the role of TBAs favourably, and appreciated the comprehensive care provided by TBAs, e.g. how TBAs accompanied women to health centres in the case of complications during home delivery:

"‘I was assisted by my mother when I gave birth, but TBAs used to hold gloves, cotton and towels. They are good at giving care and encourage women during birth. Most of the TBAs refer us to health facilities if they feel that there is a problem’. (Woman who delivered at home) - FGD 1"

### Medical knowledge under constrained circumstances

The category ‘medical knowledge under constrained circumstances’ referred to how women perceived the medical services available to them regarding delivery care. On the one hand, women realized that health facilities, especially hospitals, were resourceful at managing complications. Women appreciated the medical resources provided at health facilities in terms of human resources and non-human resources such as drugs. On the other hand, women criticized the poor quality of care provided in health facilities in this setting, both in terms of unreliability (e.g. being understaffed and under-supplied), and offering disrespectful treatment to users. The lack of reliable transportation to the health facilities and the fact that relatives were discouraged from supporting women during institutional deliveries were also negatively perceived. Four subcategories were identified in relation to this category: ‘appreciating medical resources’, ‘uncertain quality of care’, ‘transportation barriers’, ‘HEWs as gate-openers’.

#### Appreciating medical resources

Women described how their opinions had changed regarding the role of health facilities in dealing with delivery-related complications, from rejecting procedures such as caesarean sections to acknowledging that they could be life-saving interventions. Negative home delivery experiences when women faced life -threatening complications also contributed to the recognition of medical care as valuable:

"‘Nowadays hospital deliveries are becoming common and even if some problem occurs, an operation is performed and the mother and the child are kept safe without any payment for all the services provided. […] A long time ago I was afraid of operations; these days, if there seems to be a danger and if I am requested to be operated on I accept the suggestion and an operation is performed to save women’s and babies’ lives’. (Woman who delivered at health facility - FGD 6) "

Women appreciated the resources provided at the health facilities. They valued the information offered by the health workers, e.g. they described how health workers informed them about how to breast-feed their newborn babies. They felt safe and secure when assisted by skilled attendants at health facilities during delivery.

In addition they appreciated the non-human resources available at health facilities, e.g. drugs, especially injections. They valued different interventions that were applied by health workers at hospitals during pregnancy and delivery such as abdominal examination, measurement of blood pressure or checking the foetal heart beat. They also believed that giving birth at health facilities was more hygienic than at home. The fact that deliveries at health facilities were free of charge was also mentioned.

#### Uncertain quality of care

Women appreciated medical services for their ability to deal with complications and recognized the value of medical interventions in ensuring a safe delivery. Yet they also described the poor quality of the medical services available to them, partly because the services were perceived as unreliable. Women criticized health facilities for very long waiting times, and health workers for not being available during nights or weekends, and offering incomplete health services. Women also complained of the poor skills and competencies of health providers, whom they considered to be undertrained and offering inaccurate diagnoses. Health workers were criticized for not taking immediate decisions and actions when expectant mothers were not at ease; they complained about having to go back and forth to the health facility during delivery, and felt irritated for not being promptly referred to the hospital:

"‘When I gave birth to my second child I went to the health centre at night. I was bleeding and they did not refer me to hospital immediately. I spent the night there. I had severe pain, I was suffering and exhausted in a room alone, but they were watching me through the window. In between, if I die, what happens? When the grandparents and other neighbours observe, how do they feel? They would decide to stay at home; they would prefer to give birth at home. Then I went to the hospital at three o’clock in the morning. They injected me with a medicine and I gave birth immediately’. (Woman who delivered at health facility - FGD 4) "

On the other hand, women also criticized health workers' attitudes and said that they felt disrespected by them; some even insult and embarrass women during delivery.

Whenever this happened, the news spread and women were deterred from attending health facilities for delivery. Women also complained that health providers disregarded their wish for relatives to accompany them during delivery and their lack of warmth when treating mothers and newborn babies.

#### Transportation barriers

One of the reasons for women giving birth at home was because they could not accurately predict the exact time of their delivery, and consequently they could not plan their transportation to a health facility.

Participants from areas close to the town and those from remote areas described the problem of transport access in different ways. Women said that although an ambulance was available in their locality, reaching areas with difficult roads was easier said than done.

Moreover, women from remote areas mentioned a lot of complications, such as bleeding and abnormal foetal position, that could occur when expectant mothers were transported by stretcher from mountainous localities to the health facilities. They described how difficult it often was to seek transportation means during the night in a place where there were no telephones and no ambulances. Furthermore, asking neighbours to carry women on a stretcher at night was perceived as creating inconvenience. Considering the difficulties of arranging proper and timely transportation, home deliveries seemed much more convenient to the participants:

"‘I wanted to give birth at HF, but I had no one to bring me here. I could not find a car to bring me here. This is not a place like in town where you can immediately make a phone call and obtain a vehicle for transport; you have to call on a lot of people to carry you on a stretcher and take you to the HF’. (Woman who delivered at home - FGD 2)"

#### HEWs as gate-openers

This subcategory refers to the role of HEWs at the community level in creating a conducive atmosphere and harmony to support women in delivery and antenatal care services. Unlike health workers at hospitals and health centres, HEWs were nearby and known by women; they belonged to the same communities and shared similar backgrounds. For this reason, participants felt comfortable with the HEWs and appreciated it when they approached them.

"‘HEWs have a good reputation, they consider me as their sister, give me good care, they identify which specific locality I come from, they receive me with a smiling face’cxxx (Woman who delivered at health facility - FGD4) "

HEWs encouraged women to visit health facilities for antenatal care and delivery. HEWs frequently visited communities and households, and women recognized them from their engagement in different health activities. Women also appreciated how HEWs reassured expectant mothers during delivery and how they helped with referrals when complications that were beyond their capacity emerged.

The proximity of health posts to the communities has created a better opportunity for women to utilize services such as antenatal care. Participants said that the health post service was provided in a short period of time compared with health centres and hospitals, not only owing to proximity to the communities but also because of the HEWs’ commitment.

## Discussion

This study shows how women in Tigray made pragmatic choices between two models of delivery, represented respectively by the categories ‘embedded in tradition’ and ‘medical knowledge under constrained circumstances’. The model represented by ‘embedded in tradition’ has existed in Ethiopia since ancestral times, whereas the model represented by ‘medical knowledge under constrained circumstances’ is a newcomer, especially in rural parts of the country [24]. Before the existence of health facilities, there were traditional ways to deal with issues of delivery, and women were familiar with them. Moreover, despite husbands’ support for skilled birth attendants, transportation was not easily available to these women, making institutional delivery a more complex option, especially when labour started during the night or when women lived in remote areas. These two models co-existed but there was no coordination between them and there were even conflicts and competition between them.

The Ethiopian HEP builds on the principles of primary health care, and aims to network volunteer community health workers, including TBAs, with HEWs and higher-level health facilities [8]. This study shows, however, that the health system in Tigray does not seem to be aligned with this integrated approach, and the very relevant role of family and traditional agents such as TBAs in ensuring safe deliveries is diminished. HEP was designed to foster health promotion, prevention and basic curative services. During the inception of the program a strong linkage between community, TBAs, HEWs and health facility was thought to promote implementation of skilled delivery attendance and Emergency Obstetric care (EmOC). However, the local solution was not fairly integrated with the skilled attendance delivery and EmOC, due to resource and time constraints.

When the women from this study made choices, they were pragmatic. They did not see any contradiction in embracing part of one model and part of the other and tried to take the best from each model within certain limitations. Even more, within these two models, gate-openers trusted by women existed: TBAs for the first model and HEWs for the second. Albeit they advocated and promoted a different model, they were also on the side of the women, supporting them in the process of achieving a safer delivery, and not just inviting them to adopt a particular form of delivery care. These results back the continuum of care approach in considering family, traditional community agents and clinical settings not as competing actors but as collaborators [4,12].

Our results differ from other studies regarding women’s awareness of risks associated with pregnancy and delivery [25-28]. In the urban area of Tigray, one barrier to care was the lack of knowledge on danger signals, but participants in our study were well aware that delivery could place women's lives at risk [28]. Postpartum haemorrhage, the main cause of maternal mortality, was clearly understood by these women as a complication that needed urgent action. Moreover, they recognized that medical services could effectively deal with it, and did not express resistance towards measures such as emergency caesarean section, injections or blood transfusions.

Women’s appreciation of medical resources has also been identified in another region of Ethiopia [29] and could have become a strong basis for enhancing women’s access to skilled delivery attendance and emergency obstetric care services. In Tigray, however, the dismal situation of maternal healthcare services posed an enormous barrier for women actually using these services during delivery. If enhancing quality of care regarding availability of drugs or technical skills is expensive and hard to achieve in resource-limited settings, respectful interactions with users do not need high investment but rather an attitudinal change in the institutional culture of the health systems [25,30,31]. Previous studies in Ethiopia have also stated the importance of provider-patient interaction in ensuring adequate service delivery [31]. Our study also points out the importance of allowing relatives and/or community health care workers into the delivery room, another change that would increase women’s acceptance of institutional delivery and has proven to be cost-effective [32].

Results from this study align with previous research from similar settings which also points out that transportation difficulty remains a barrier to accessing institutional delivery [33-36]. This study points out that even if relatives and neighbours are willing to transport women using stretchers, it is not seen as convenient or safe by women. Other barriers, such as embarrassment or fear of causing inconvenience, prevented women from accessing a health facility, especially considering that women were well aware that when they reached the services resources might not be available or the quality might not be good.

This study stresses the important role of health workers at the community level, both traditional health workers such as TBAs and health workers integrated within the health systems such as HEWs. Other studies in Ethiopia have highlighted the important role that HEWs play [37]. Women felt them to be closer, perceived them as more reliable, and observed that their relationship with them was more respectful. Women also said that they would like to have access to higher resolution services provided by HEWs at the level of the *kebele*. Offering emergency obstetric services at this level is currently unaffordable, however, and consequently a good referral system is necessary. It means that community workers should be in close contact with higher resolution facilities, in order to ensure an adequate response to problems and complications that may arise during pregnancy, delivery and postpartum.

Another interesting finding emerging from this study was the positive perception women had regarding the involvement of their husbands during delivery. Other studies in Ethiopia have pointed out the key role of husbands in decision-making regarding seeking health care during delivery. They portrayed husbands mainly as barriers to their wives’ health, however, e.g. they were unwilling to spend money on their wives' well-being and preferred to wait when complications emerged [8,38,39].

### Methodological considerations

The setting of the FGDs where women were interviewed and the first author’s position as a member of the Medical Faculty of the University may have affected participants’ opinions through social desirability bias. The fact that the first author was a man, interviewing women could have affected the results, albeit during the FGDs women seemed to participate actively and freely. Another limitation of the study relates to the fact that the interviews were translated into English for the analysis, allowing the involvement of all four researchers. This could have led to some of the original meanings narrated by the participant women in Tigray being lost.

Participants were purposely selected for their ability to contribute to the research question, and an effort was made to contextualize the results and to detail the analytical process. However, the way informants were selected by HEWs could have led to social desirability bias, namely respondents providing a more positive attitude towards institutional delivery.

It would have been useful to collect information from other key actors on delivery (such as TBAs or HEWs) but here we decided to focus on women’s perspectives, and how they perceived the roles of delivery attendants, both professionals and TBAs.

Measures were taken to strengthen trustworthiness. In order to enhance dependability, an emergent design was followed and the guide with questions incorporated relevant issues that emerged from previous FGDs. Since the first author was originally from Tigray and living in the area, credibility – how well the findings had captured the reality being explored was enhanced by prolonged engagement. The fact that the other researchers were not familiar with the setting added the external perspective. Triangulation of researchers also contributed to credibility [40].

## Conclusions

The women who participated in this study were actively making choices for a safer delivery. In this process they had to choose between two models of childbirth, the traditional model represented by the category ‘embedded in tradition’ and the medical model represented by the category ‘medical knowledge under constrained circumstances’.

Women were pragmatic in their decisions, and did not feel any conflict when choosing between the two available models of delivery. These two models, however, were not as open as the women themselves. Women had to decide between (1) giving birth at home, in a known place, sanctioned by tradition, faith and the ascendancy of the elderly, but where little could be done to manage complications, or (2) delivering at health facilities, where medical knowledge could (theoretically) manage complications, but where services’ availability was on occasion erratic, the quality of care poor and staff’s attitudes disrespectful. Women were not alone in their struggle for a safe delivery: traditional birth attendants, health extension workers, and husbands were perceived as sharing the women's concerns.

This study indicates that debates regarding effective interventions to decrease maternal mortality should not be reduced to theoretical efficiency but should take into account that contextual factors shape the way in which these interventions are actually implemented in real life. The current low rate of skilled birth attendants both in Tigray and nationally indicates that the balance of decisions favours the ‘traditional model’. Women have however expressed a positive opinion about the ‘medical model’ but this demands a stronger commitment from the healthcare authorities. We suggest that health services should increase their availability and quality of care, the TBAs' role should be re-examined, and training programmes strengthened. Moreover, we recommend that a well-designed, coordinated and feasible referral system should be implemented. Along with these, health institutions should allow a community care giver or relative during child birth for provision of continous emotional support to women which women considered as a key factor for satisfying their needs.

## Competing interests

The authors declare that they have no competing interests.

## Authors' contributions

TG designed the study, participated in data collection and analysis and prepared the draft of the manuscript; IG, KE and MSS provided scientific advices on the design of the study, data analysis and throughout the preparation of the manuscript. All authors read and approved the final manuscript.

## Pre-publication history

The pre-publication history for this paper can be accessed here:

http://www.biomedcentral.com/1471-2393/12/113/prepub

## References

[B1] Nations U The Millennium Development Goals 2010 New York: United Nations Report

[B2] Hill K Thomas K AbouZhar C Walker N Say L Inoue M Suzuki E Estimates of maternal mortality worldwide between 1990 and 2005: an assessment of available data Lancet 2007 370 9595 1311 1319 10.1016/S0140-6736(07)61572-4 17933645

[B3] Ronsmans C Graham WJ Maternal mortality: who, when, where, and why Lancet 2006 368 9542 1189 1200 10.1016/S0140-6736(06)69380-X 17011946

[B4] Lawn JE Tinker A Munjanja SP Cousens S Where is maternal and child health now? Lancet 2006 368 9546 1474 1477 10.1016/S0140-6736(06)69387-2 17071267

[B5] Kinney MV Kerber KJ Black RE Cohen B Nkrumah F Coovadia H Nampala PH Lawn JE Sub-Saharan Africa's Mothers, Newborns, and Children: Where and Why Do They Die? PLoS Med 2010 7 6 e1000294 10.1371/journal.pmed.1000294 20574524 PMC2888581

[B6] Darmstadt GL Lee AC Cousens S Sibley L Bhutta ZA Donnay F Osrin D Bang A Kumar V Wall SN Baqui A Lawn JE 60 million non-facility births: who can deliver in community settings to reduce intra-partum related deaths? Int J Gynaecol Obstet 2009 107 S89 S112 19815200 10.1016/j.ijgo.2009.07.010 PMC3428830

[B7] Greenwood AM Bradley AK Byass P Greenwood BM Snow RW Bennett S Hatib-N’Jie AB Evaluation of a primary health care programme in the Gambia. I. The impact of trained traditional birth attendants on the outcome of pregnancy J Trop Med Hyg 1990 93 1 58 66 2304134

[B8] Koblinsky M Tain F Gaym A Responding to the maternal health care challenge: The Ethiopian Health Extension Program Ethiop J Health Dev 2010 24 1 105 109

[B9] Freedman LP Graham WJ Brazier E Smith JM Ensor T Fauveau V Themmen E Currie S Agarwal K Practical lessons from global safe motherhood initiatives: time for a new focus on implementation Lancet 2007 370 9595 1383 1391 10.1016/S0140-6736(07)61581-5 17933654

[B10] Yosuf J Mulatu T Nigatu T Revisiting the exclusion of TBAs from formal health systems in Ethiopia 2010 Kenya-Nairobi: AMREF

[B11] Nyamtema AS Urassa DP van Roosmalen J Maternal health interventions in resource limited countries: a systematic review of packages, impacts and factors for change BMC Pregnancy Childbirth 2011 11 30 10.1186/1471-2393-11-30 21496315 PMC3090370

[B12] Kerber KJ de Graft-Johnson JE Bhutta ZA Okong P Starrs A Lawn JE Continuum of care for maternal, newborn, and child health: from slogan to service delivery Lancet 2007 370 9595 1358 1369 10.1016/S0140-6736(07)61578-5 17933651

[B13] WHO The partnership for maternal, newborn and child health 2011 WHO: Geneve Available from: [http://www.who.int/pmnch/en/], [accessed on 2011 May 20].

[B14] McCoy D Storeng K Filippi V Ronsmans C Osrina D Matthiasb B Campbell OM Wolfeb R Prosta A Hilla Z Costelloa A Azadc K Manandhare DSM Maternal, neonatal and child health interventions and services: moving from knowledge of what works to systems that deliver International Health 2010 2 3 87 98 10.1016/j.inhe.2010.03.005 24037704

[B15] Accorsi S Bilal NK Farese P Racalbuto V Countdown to 2015: comparing progress towards the achievement of the health Millennium Development Goals in Ethiopia and other sub-Saharan African countries Trans R Soc Trop Med Hyg 2010 104 5 336 342 10.1016/j.trstmh.2009.12.009 20106495

[B16] Federal Ministry of Health Health extension programme implementation guidelines 2004 Addis Ababa: Ministry of Health

[B17] Federal Ministry of Health Health extension programme in Ethiopia 2007 Addis Ababa: Ministry of Health

[B18] Federal Ministry of Health Health sector development programme, HSDPIII, 2005/06 – 2010/11, mid-term review, volume I 2008 Addis Ababa: Ministry of Health

[B19] Karim AM Betemariam W Yalew S Alemu H Carnell M Mekonnen Y Programmatic correlates of maternal healthcare seeking behaviors in Ethiopia Ethiop J Health Dev 2010 24 1 92 99

[B20] Tsegay Y Determinants of Antenatal Care, Institutional Delivery and Skilled birth Attendant Utilization in Ethiopia MPH thesis 2010 Umea, Sweden: Department of Public Health and Clinical Medicine; Umea University

[B21] Tigray Health Bureau Tigray Health Profile 2007/2008 2008 Mekelle: Tigray Health Bureau

[B22] Tigray Health Bureau Tigray Health Profile 2010 2010 Mekelle: Tigray Health Bureau

[B23] Dhalgren L Emmelin M Winkvist A Qualitative Methodology for International Public Health 2004 Umeå, Sweden: Umeå University

[B24] Gidey G Tagu S Hagos S Lecture notes for health science students, Introduction to Public Health: Chapter four, Traditional health care practices EPHTI, USAID, Addis Ababa, Ethiopia 2006

[B25] Jennings L Yebadokpo AS Affo J Agbogbe M Antenatal counseling in maternal and newborn care: use of job aids to improve health worker performance and maternal understanding in Benin BMC Pregnancy Childbirth 2010 10 75 10.1186/1471-2393-10-75 21092183 PMC3002891

[B26] Mutiso SM Qureshi Z Kinuthia J Birth preparedness among antenatal clients East Afr Med J 2008 85 6 275 283 18817024 10.4314/eamj.v85i6.9625

[B27] Turan JM Tesfagiorghis M Polan ML Evaluation of a community intervention for promotion of safe motherhood in Eritrea J Midwifery Womens Health 2011 56 1 8 17 10.1111/j.1542-2011.2010.00001.x 21323845 PMC3498940

[B28] Hiluf M Fantahun M Birth preparedness and complication readiness among women in Adigrat town, north Ethiopia Ethiop J Health Dev 2007 22 1 14 20

[B29] Kruk ME Paczkowski MM Tegegn A Tessema F Hadley C Asefa M Galea S Women's preferences for obstetric care in rural Ethiopia: a population-based discrete choice experiment in a region with low rates of facility delivery J Epidemiol Community Health 2010 64 11 984 988 10.1136/jech.2009.087973 19822558

[B30] Van Eijk AM Bles MH Odhiambo F Ayisi JG Blokland IE Rosen DH Adazu K Slutsker L Lindblade KA Use of antenatal services and delivery care among women in rural western Kenya: a community based survey Reprod Health 2006 3 2 10.1186/1742-4755-3-2 16597344 PMC1459114

[B31] Birhanu Z Assefa T Woldie M Morankar S Determinants of satisfaction with health care provider interactions at health centres in central Ethiopia: a cross sectional study BMC Health Serv Res 2010 10 78 10.1186/1472-6963-10-78 20334649 PMC2848139

[B32] Mrisho M Obrist B Schellenberg JA Haws RA Mushi AK Mshinda H Tanner M Schellenberg D The use of antenatal and postnatal care: perspectives and experiences of women and health care providers in rural southern Tanzania BMC Pregnancy Childbirth 2009 9 10 10.1186/1471-2393-9-10 19261181 PMC2664785

[B33] Onah HE Ikeako LC Iloabachie GC Factors associated with the use of maternity services in Enugu, south eastern Nigeria Soc Sci Med 2006 63 7 1870 1878 10.1016/j.socscimed.2006.04.019 16766107

[B34] Kiwanuka SN Ekirapa EK Peterson S Okui O Rahman MH Peters D Pariyo GW Access to and utilization of health services for the poor in Uganda: a systematic review of available evidence Trans R Soc Trop Med Hyg 2008 102 11 1067 1074 10.1016/j.trstmh.2008.04.023 18565559

[B35] Gabrysch S Campbell OM Still too far to walk: Literature review of the determinants of delivery service use BMC pregnancy child birth 2009 9 34 10.1186/1471-2393-9-34 PMC2744662 19671156

[B36] Negusse H McAuliffe E MacLachlan M Initial community perspectives on the Health Service Extension Programme in Welkait Ethiopia. Hum Resour Health 2007 5 21 10.1186/1478-4491-5-21 PMC2000465 17718900

[B37] Warren C Care seeking for maternal health: challenges remain for poor women Ethiop J Health Dev 2010 24 1 100 104

[B38] Berhane Y Gossaye Y Emmelin M Högberg U Women’s health in rural setting in societal transition in Ethiopia Soc Sci Med 2001 53 11 1525 1539 10.1016/S0277-9536(00)00441-X 11710427

[B39] Lincoln Y Guba E Naturalistic Inquiry 1985 New York: SAGE

[B40] Van Roosmalen J Walraven G Stekelenburg J Massawe S Integrating continuous support of the traditional birth attendant into obstetric care by skilled midwives and doctors: a cost-effective strategy to reduce perinatal mortality and unnecessary obstetric interventions Trop Med Int Health 2005 10 5 393 394 10.1111/j.1365-3156.2005.01411.x 15860084

